# Supplementary material for: CUSP: Complex spike sorting from multi-electrode array recordings with U-net sequence-to-sequence prediction
Source: J Neurosci Methods. Author manuscript; Available in PMC 2026 Jan 2. (PMC12757777; doi:10.1016/j.jneumeth.2025.110631)
Supplement: MMC1 [file NIHMS2125861-supplement-MMC1.pdf]

Fig. S1

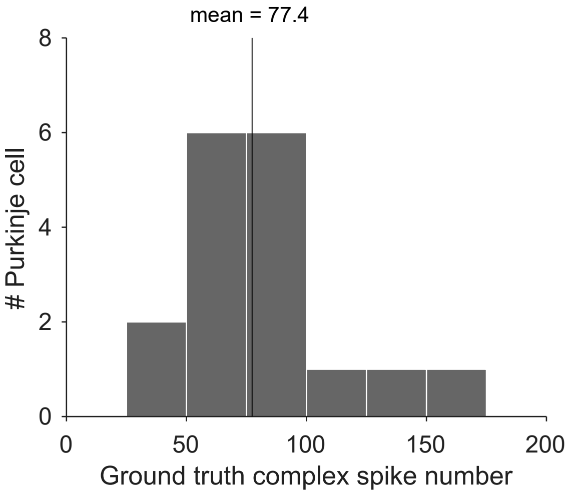
mean = 77.4
# Purkinje cell
0
2
4
6
8
0
50
100
150
200
Ground truth complex spike number

Fig. S2

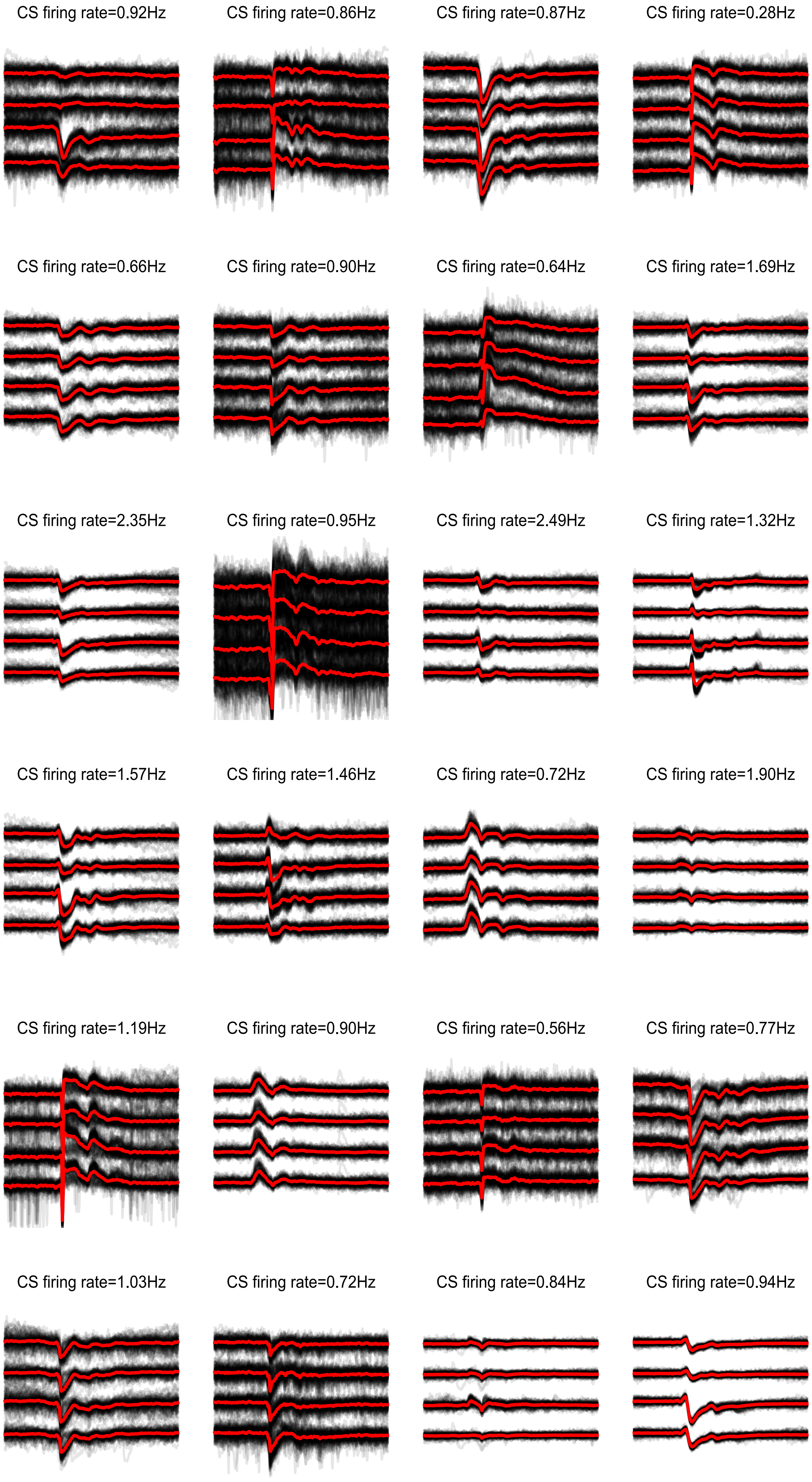

CS firing rate=0.92Hz
CS firing rate=0.86Hz
CS firing rate=0.87Hz
CS firing rate=0.28Hz
CS firing rate=0.66Hz
CS firing rate=0.90Hz
CS firing rate=0.64Hz
CS firing rate=1.69Hz
CS firing rate=2.35Hz
CS firing rate=0.95Hz
CS firing rate=2.49Hz
CS firing rate=1.32Hz
CS firing rate=1.57Hz
CS firing rate=1.46Hz
CS firing rate=0.72Hz
CS firing rate=1.90Hz
CS firing rate=1.19Hz
CS firing rate=0.90Hz
CS firing rate=0.56Hz
CS firing rate=0.77Hz
CS firing rate=1.03Hz
CS firing rate=0.72Hz
CS firing rate=0.84Hz
CS firing rate=0.94Hz

Fig. S3

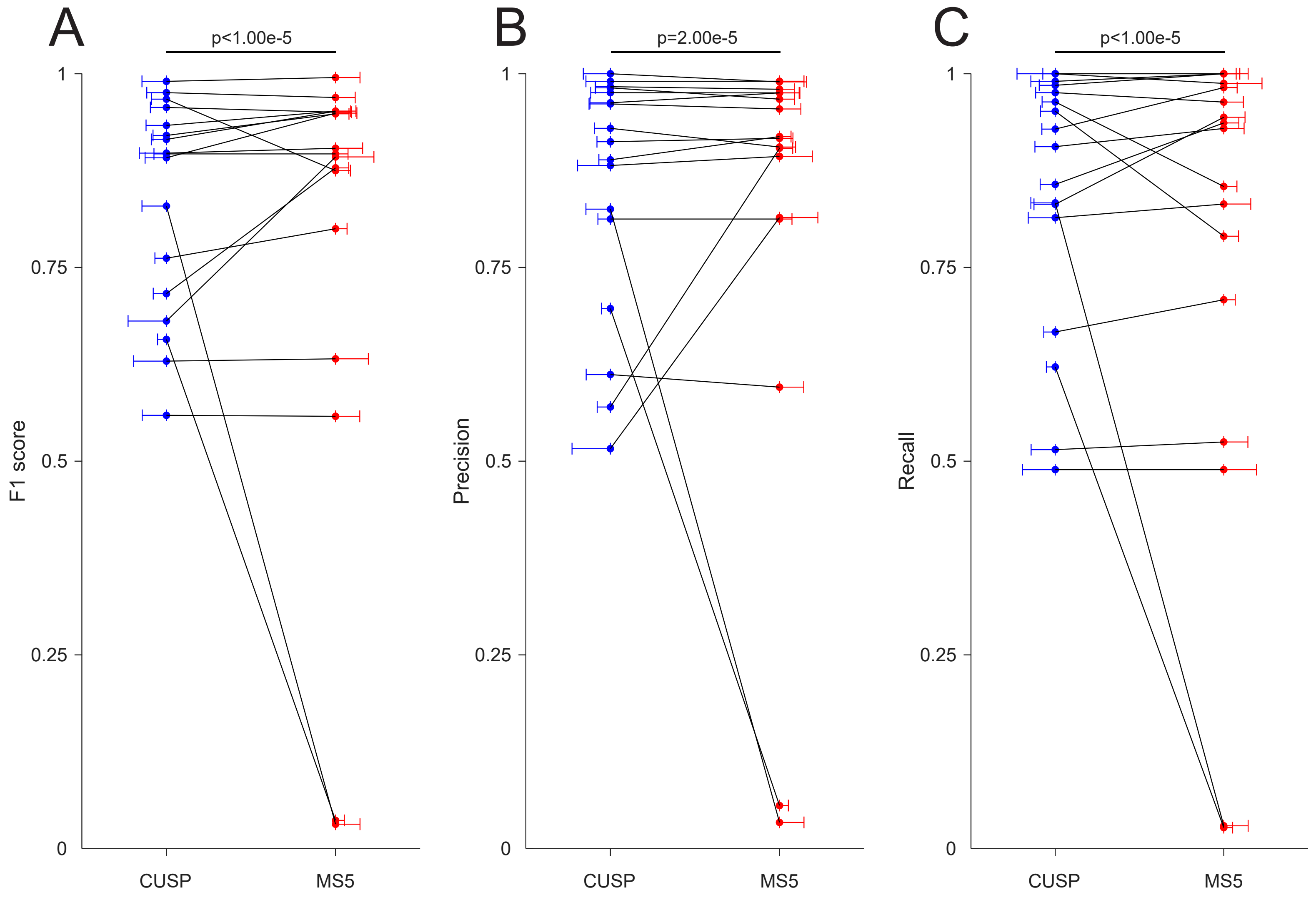

A
p<1.00e-5
F1 score
1
0.75
0.5
0.25
0
CUSP
MS5
B
p=2.00e-5
Precision
1
0.75
0.5
0.25
0
CUSP
MS5
C
p<1.00e-5
Recall
1
0.75
0.5
0.25
0
CUSP
MS5

Fig. S4

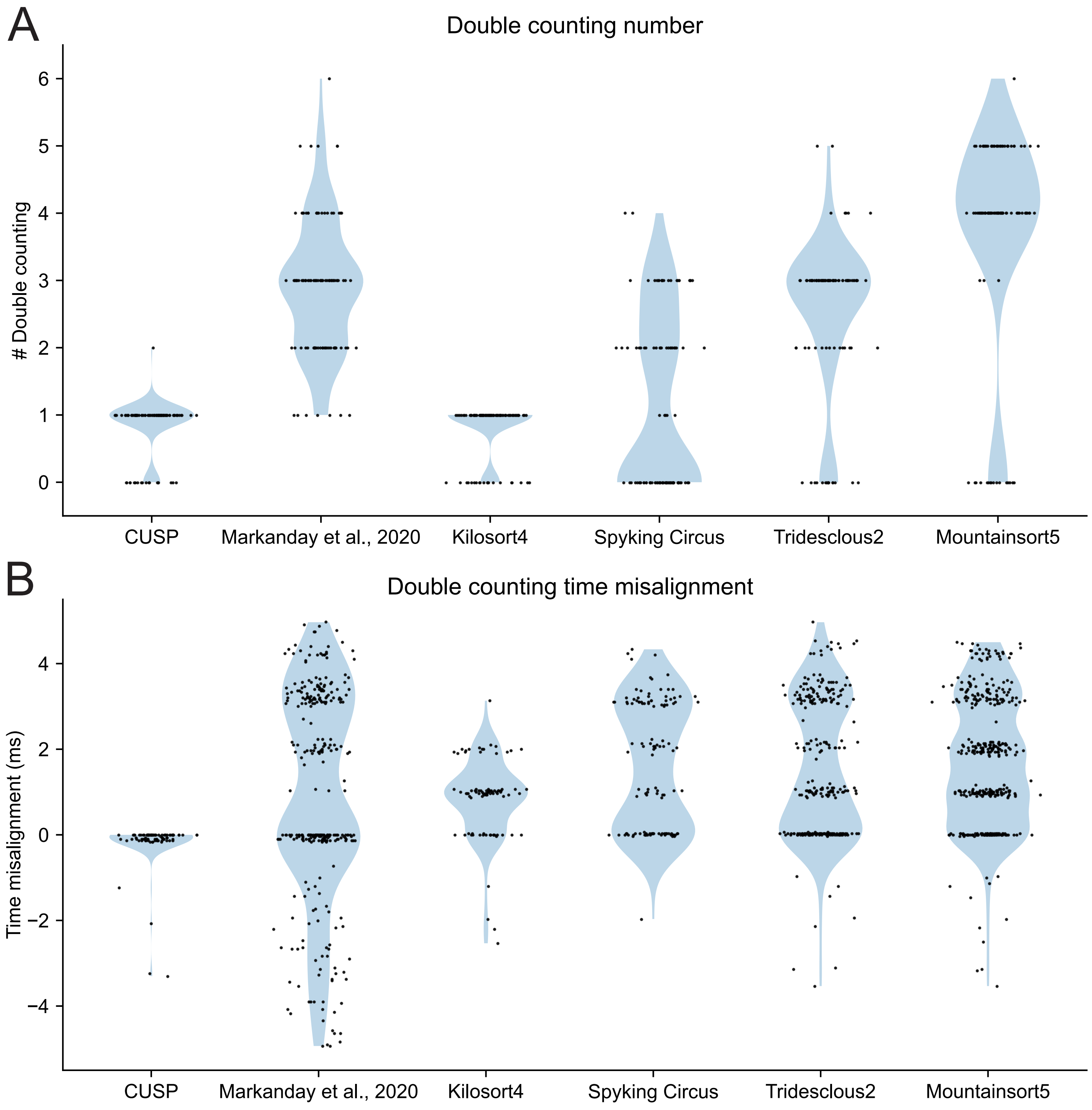
A
Double counting number
Double counting
0
1
2
3
4
5
6
CUSP
Markanday et al., 2020
Kilosort4
Spyking Circus
Tridesclous2
Mountainsort5
B
Double counting time misalignment
Time misalignment (ms)
−4
−2
0
2
4
CUSP
Markanday et al., 2020
Kilosort4
Spyking Circus
Tridesclous2
Mountainsort5

Fig. S5

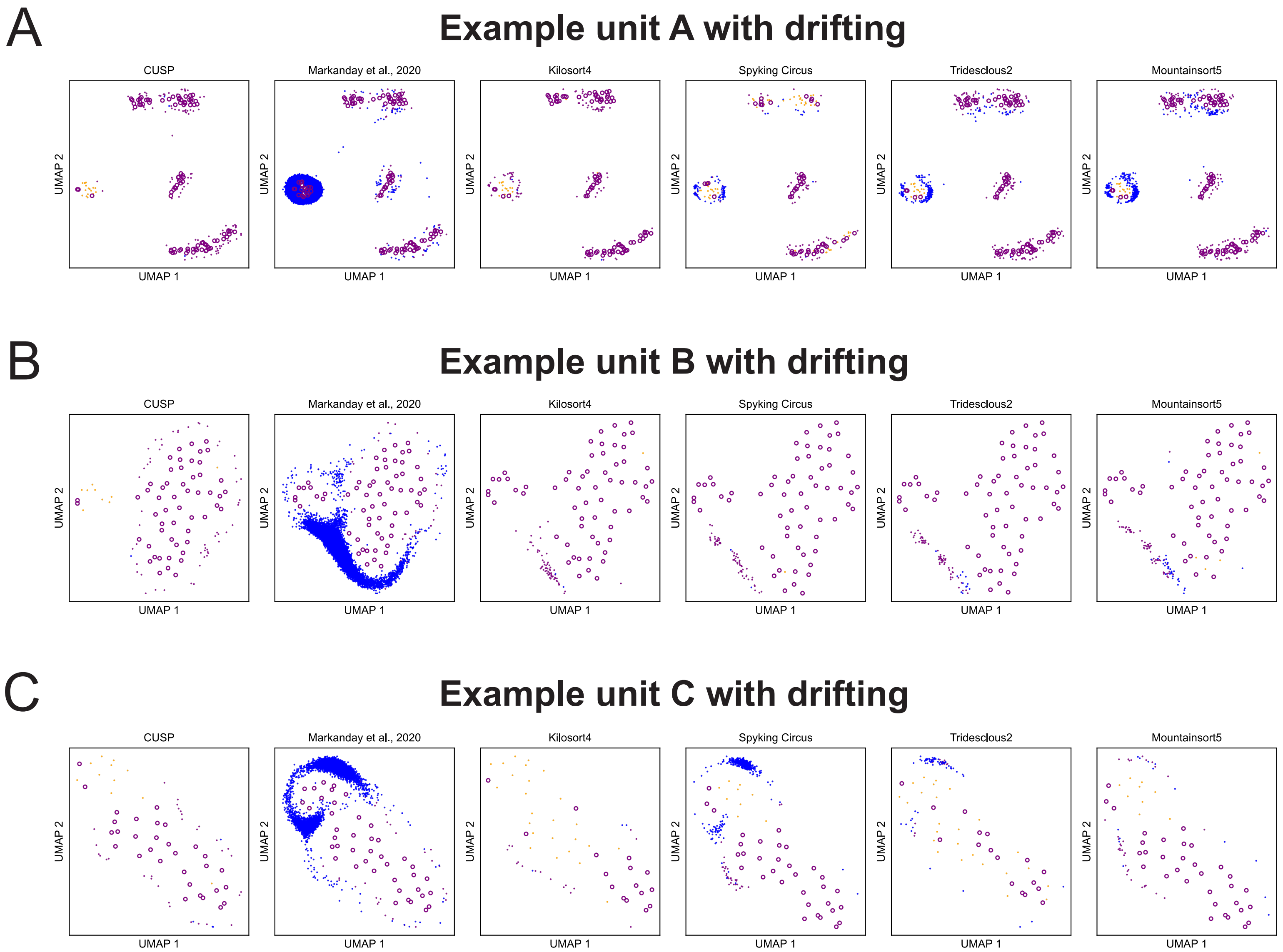
A
Example unit A with drifting
B
Example unit B with drifting
C
Example unit C with drifting
CUSP
Markanday et al., 2020
Kilosort4
Spyking Circus
Tridesclous2
Mountainsort5
UMAP 1
UMAP 2
