# Supplementary material for: CUSP: Complex spike sorting from multi-electrode array recordings with U-net sequence-to-sequence prediction
Source: J Neurosci Methods. Author manuscript; Available in PMC 2026 Jan 2. (PMC12757777; doi:10.1016/j.jneumeth.2025.110631)
Supplement: MMC2 [file NIHMS2125861-supplement-MMC2.docx]

**Fig. S1. Histogram of ground truth expert labeled complex spike number per number in our dataset.** In average, leaving out 1 Purkinje cells will exclude 77.4 ground truth expert labeled CS event instances from training.

**Fig. S2. CUSP-detected complex spike examples from recording database outside the reported expert-labeled complex spike datasets.** Each complex spike example contains four-channel waveforms adjacent to the complex spike peak channel (black), the averaged waveform (red), and its averaged firing rate at resting discharge.

**Fig. S3. CUSP shows significantly higher complex spike sorting performance than the existing state-of-the-art spike sorter.** Paired permutation tests were performed between CUSP and the existing state-of-the-art spike sorter (Mountainsort5) to compare **(A)** F1 scores, **(B)** precision scores, and **(C)** recall scores during the leave-one-out cross validation. The randomized permutations were re-weighted based on the total expert labeled complex spike numbers in each session to compensate for the imbalance among the variable session time lengths and complex spike firing rates and then repeated for 100,000 times. Each black line is one session and each dot is the score value of CUSP (blue) or Mountainsort5 (red). The length of the horizontal bar near each dot is proportion to the permutation weight (total expert labeled complex spike number) in that session. F1 score: p<1.00e-5; precision score: p=2.00e-5; recall score: p<1.00e-5.

**Fig. S4. CUSP shows less double-counting problems when sorting complex spikes with spikelet diversity.** **(A)** Double-counting number counting for each algorithm in an example session when sorting complex spikes with variable spikelet numbers and inter-spikelet intervals. Each black dot is the multiple-counting number of each algorithm detections for each expert-labeled complex spike. The blue area shows the general double counting number distributions for each algorithm. **(B)** Double-counting time misalignment for each algorithm in the same example session. Each black dot is the time misalignment of each algorithm detection relative to the expert-labeled complex spike onset time. The blue area shows the general double counting time misalignment distributions for each algorithm.

**Fig. S5. CUSP shows its detected complex spike cluster distributions aligned with the manifold of the expert labeled complex spikes in the embedded space.** **(A-C)** The expert-labeled complex spike waveforms from three example units with recording drifting were embedded into two-dimensional manifold using UMAP, as well as with each algorithm detected complex spike waveforms projected into the same two-dimensional space using UMAP. The purple circle indicates each successfully detected expert-labeled complex spike; the purple dot indicates each algorithm detected complex spike that was matched to an expert-labeled complex spike; the blue dot indicates complex spike labeled only by each algorithm; the yellow dot indicates the complex spike labeled only by experts.
